# 859. Suboptimal Uptake, Retention, and Adherence of Daily Oral PrEP Among People with OUD Receiving HCV Treatment

**DOI:** 10.1093/ofid/ofab466.1054

**Published:** 2021-12-04

**Authors:** Christopher J Brokus, Jasmine Stevens, Rachel Silk, Julia Mount, Catherine Gannon, Rahwa Eyasu, Ashley Davis, Amelia Cover, Emade Ebah, Britt Gayle, Onyinyechi Ogbumbadiugha-Weekes, Shivakumar Narayanan, Phyllis Bijole, Miriam Jones, Randy Kier, David Sternberg, Henry Masur, Shyam Kottilil, Sarah Kattakuzhy, Elana S Rosenthal

**Affiliations:** 1 University of Maryland School of Medicine, Boston, MA; 2 National Institutes of Health, Baltimore, MD; 3 University of Maryland, Washington, DC; 4 Institute of Human Virology, University of Maryland School of Medicine, Balltimore, MD; 5 HIPS, Washington, DC

## Abstract

**Background:**

Daily oral pre-exposure prophylaxis (PrEP) with tenofovir/emtricitabine (TDF/FTC) effectively prevents HIV among people who use drugs (PWUD). Despite rising rates of HIV incidence and injection drug use, PrEP use remains low and limited research exists on PrEP adherence and retention in this population.

**Methods:**

Based in Washington, DC and Baltimore, the ANCHOR investigation evaluated a community-based model of care collocating hepatitis C (HCV) therapy, medication for opioid use disorder (OUD), and PrEP in people with chronic HCV, OUD, and drug use within 1 year. PrEP counseling was offered from HCV treatment Day 0 until Week 24 and subjects could start any time during this window. PrEP patients were followed for 48 weeks and assessed for adherence by self-report and dried blood spot analysis of TDF.

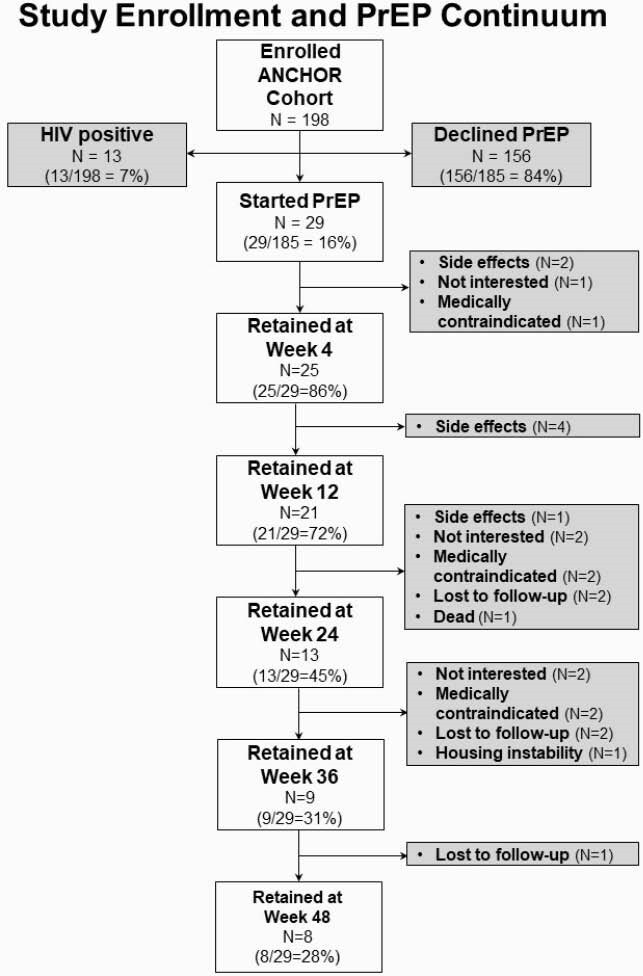

ANCHOR PrEP study enrollment and participant retention along the PrEP continuum.

**Results:**

198 participants enrolled in ANCHOR, of whom 185 (93%) were HIV-negative. 29 subjects (16% of HIV-negative group) initiated PrEP. 116 subjects (63%) met 2014 CDC criteria for PrEP initiation due to IDU (82, 44%), sex (9, 5%), or both (25, 14%). Those who initiated were more likely to meet both CDC sexual and IDU risk criteria than those who declined PrEP (P=0.006). Providers recommended PrEP to 94 subjects (51%), which was associated with uptake (P=0.02). While median treatment duration was 104 days (IQR 28, 276), only 8 subjects were retained through Week 48. The most common reason for discontinuation was side effects in 7 subjects or 24% of PrEP subgroup. Treatment interruptions occurred in one-third of the PrEP subgroup. Adherence of 4 to 7 pills per week was variable over time by self-report and declined by TDF analysis. No HIV seroconversions occurred.

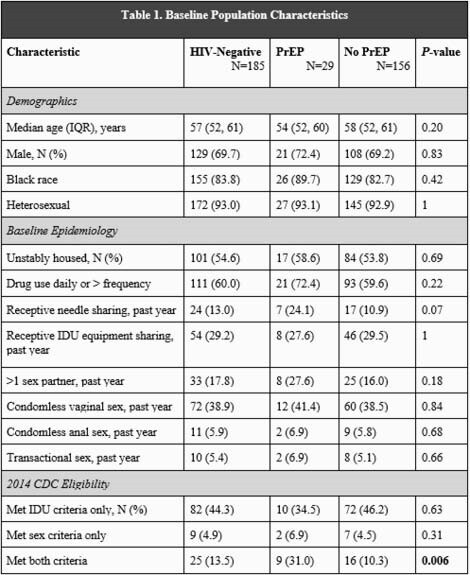

Demographic and epidemiological background of the ANCHOR study population.

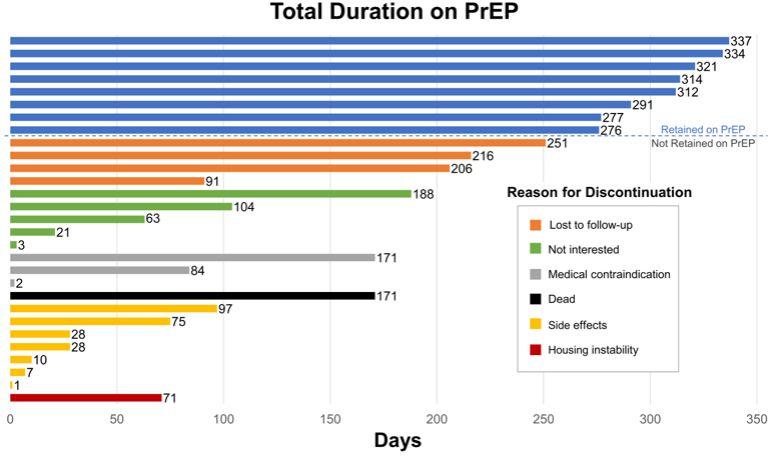

Total duration, in days, on PrEP in the ANCHOR study. Discontinued participants are grouped by reason for cessation of therapy.

PrEP Adherence

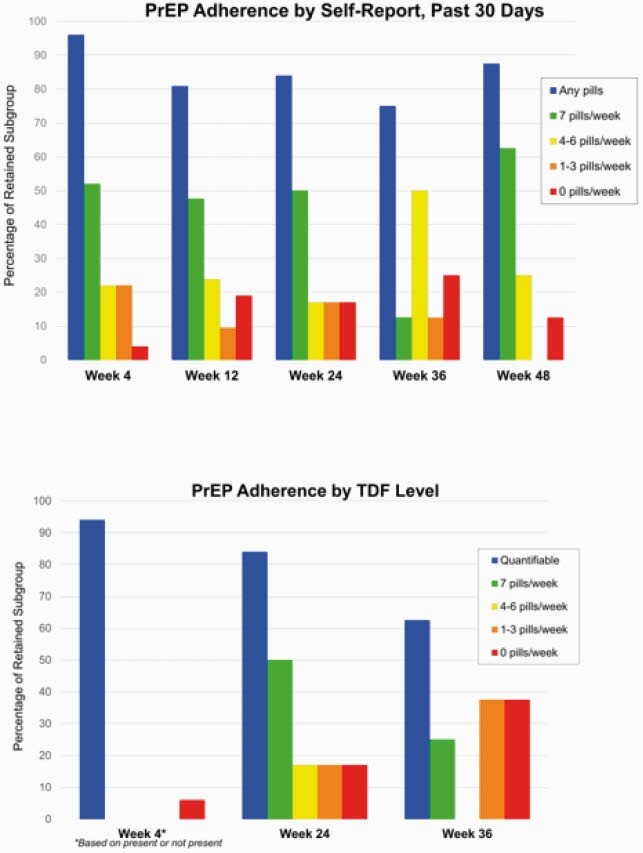

Adherence to PrEP by ANCHOR study timepoint, assessed via self-report (above) and dried bloodspot analysis of tenofovir level (below).

**Conclusion:**

In this cohort of people with OUD and HCV, 16% of subjects started PrEP. While clinical recommendation was associated with uptake, high rates of disruption and discontinuation, compounded by variable pill adherence, made daily oral TDF/FTC a suboptimal preventive strategy in this cohort. Emerging PrEP modalities like long-acting injectables have potential to address these barriers, but PWUD have been excluded from their research and development to date. Additional work to identify vulnerable individuals and to promote use, adherence, and retention will be critical in implementing PrEP more effectively in this key population.

**Disclosures:**

**Sarah Kattakuzhy, MD**, **Gilead Sciences** (Scientific Research Study Investigator, Research Grant or Support) **Elana S. Rosenthal, MD**, **Gilead Sciences** (Research Grant or Support)**Merck** (Research Grant or Support)

